# Acupuncture combined with repetitive transcranial magnetic stimulation for the treatment of post-stroke depression: a systematic evaluation and meta-analysis based on a randomised controlled trial

**DOI:** 10.3389/fneur.2024.1360437

**Published:** 2024-05-16

**Authors:** Keyuan Xiao, Xiang Li, Wenqing Hu, Xinghua Li

**Affiliations:** Changzhi People’s Hospital Affiliated to Changzhi Medical College, Changzhi, China

**Keywords:** acupuncture, repetitive transcranial magnetic stimulation, post-stroke depression, systematic review, meta-analysis

## Abstract

**Objective:**

This study aimed to systematically assess the efficacy of combining acupuncture with repetitive transcranial magnetic stimulation (rTMS) in treating post-stroke depression (PSD).

**Methods:**

We conducted a comprehensive search of eight major domestic and international databases, including the China Knowledge Network, from inception until December 2023. Included were randomized controlled trials that investigated acupuncture combined with rTMS for PSD. The screening process adhered to predetermined inclusion and exclusion criteria, and study quality was assessed using Cochrane Handbook 5.1 guidelines. Meta-analysis was conducted using RevMan 5.4 software.

**Results:**

Twelve studies involving 800 patients were included in the analysis. The meta-analysis showed that acupuncture combined with rTMS significantly improved the clinical effectiveness rate (RR = 1.19, 95% CI: 1.12 to 1.27, *p* < 0.00001) and reduced scores on several scales: Hamilton Depression Scale (HAMD) (MD = −3.35, 95% CI: −3.79 to −2.90, *p* < 0.00001), Self-Depression Scale (SDS) (MD = −9.57, 95% CI: −12.26 to −6.89, *p* < 0.00001), Chinese Medicine Symptom Score (MD = −3.34, 95% CI: −3.76 to −2.91, *p* < 0.00001), Pittsburgh Sleep Quality Scale (MD = −3.91, 95% CI: −4.58 to −3.25, *p* < 0.00001), and National Institutes of Health Stroke Scale (NIHSS) (MD = −2.77, 95% CI: −3.21 to −2.32, *p* < 0.00001). Furthermore, acupuncture combined with rTMS treatment improved cognitive functioning (MMSE, MoCA scores) (*p* < 0.00001) and ability to perform activities of daily living scores (MD = 10.40, 95% CI: 9.53 to 11.28, *p* < 0.00001). Additionally, it was found to reduce interleukin 6, tumor necrosis factor alpha, interleukin 1β, and increase 5-hydroxytryptamine and brain-derived neurotrophic factor levels (*p* < 0.001).

**Conclusion:**

Acupuncture combined with rTMS therapy is recommended for treating PSD, as it effectively improves clinical outcomes, alleviates depressive symptoms, enhances cognitive function, and daily living capabilities, and modulates inflammatory responses and neurotransmitter levels. However, it is important to note that the limitations of the sample size and quality of the included studies warrant the need for more high-quality research to validate these conclusions.

**Systematic review registration:**

INPLASY, Identifier INPLASY202430085.

## 1 Introduction

Post-stroke depression (PSD) is a psychiatric disorder that often arises after a stroke, affecting even those without any prior mental health history. It is a frequent complication (1). A comprehensive global epidemiological survey indicates that PSD prevalence ranges from 17 to 34.3% (2). The mechanisms underlying PSD remain unclear, though proposed theories include neurobiological aspects like the stroke focal mechanism hypothesis, neurotransmitter disruptions, reduced cerebral trophic factor activity, glutamate toxicity, and psychosocial factors (3).

The limited understanding of these mechanisms complicates the development of effective PSD treatments, maintaining a high incidence rate of around 30% (4). In Western medicine, the primary approach to treatment involves the use of antidepressant medications, such as selective serotonin reuptake inhibitors (SSRIs), selective serotonin and norepinephrine reuptake inhibitors (SNRIs), norepinephrine and specific serotonin antidepressants (NaSSAs), and tricyclic antidepressants. However, these medications often have significant side effects, a slow onset of action, a long treatment duration, and are ineffective in approximately one-third of patients (5). Additionally, non-pharmacological treatments such as electroconvulsive therapy (ECT), transcranial direct current stimulation (tDCS), transcranial alternating current stimulation (tACS), and repetitive transcranial magnetic stimulation (rTMS) are available (6). ECT, effective for refractory depression, includes non-convulsive and conventional forms. It involves the administration of brief, moderate electrical currents to the brain to induce temporary loss of consciousness or generalized convulsions, thereby alleviating psychiatric symptoms (7, 8). tDCS, a growing neuromodulation treatment for depression, applies mild electrical stimulation via scalp electrodes to modulate neural connectivity, cortical excitability, cerebral blood flow, and synaptic plasticity for antidepressant effects (9). tACS, avoiding sensory stimulation, potentially improves patient adherence compared to traditional techniques (10). The efficacy of traditional tDCS for treating depressive disorders is influenced by various factors, including the stimulation location (11, 12), intensity, duration, frequency (13), and combination with medication (14).

rTMS is a preferred, non-invasive, and clinically proven treatment for PSD (15). However, the use of rTMS therapy alone for PSD has certain limitations, given the chronic and recurrent nature of depression. Therefore, combining rTMS therapy with other treatment approaches has been clinically explored and found to be more effective (16). Acupuncture and moxibustion, traditional Chinese treatments, have a long history and have shown significant efficacy in treating PSD in China (17). As a safe and non-toxic alternative, acupuncture is gaining global recognition. This study aims to evaluate systematically the efficacy of combining acupuncture with rTMS in treating PSD through a comprehensive literature review. This study’s findings aim to offer valuable insights into treating PSD.

## 2 Methods

This systematic review and meta-analysis was conducted in strict adherence to the PRISMA guidelines (18). The study was duly registered with INPLASY (Registration ID INPLASY202430085), serving as an international prospective registry for systematic reviews (refer to Supplementary Table S1 for comprehensive details).

### 2.1 Literature search strategy

An exhaustive electronic search was executed across a range of databases including the China Knowledge Network (CNKI), Wanfang Database, VIP Database, PubMed, Embase, Cochrane Library, and China Biology Medicine disc (CBMD). The objective was to identify relevant randomized controlled trials (RCTs) that investigate the efficacy of acupuncture in conjunction with rTMS for the treatment of PSD. The search was conducted in both Chinese and English, spanning from the inception of the databases to December 2023. The methodology combined subject-specific terms with free-text keywords. Search terms for Chinese databases included “stroke,” “cerebral infarction,” “cerebral hemorrhage,” “post-stroke depression,” “depression,” “repetitive transcranial magnetic stimulation,” and “acupuncture.” Corresponding English terms were utilized similarly. Supplementary Table S1 detail the employed search strategies.

### 2.2 Literature inclusion criteria

(1) Study Subjects: inclusion criteria were based on standards prescribed by the Chinese Association of Cerebrovascular Diseases (19). Participants included patients diagnosed with cerebral hemorrhage or cerebral infarction verified by CT imaging. No restrictions were placed on age, gender, disease duration, or ethnicity, provided the baseline data were analogous. Diagnostic criteria from the Chinese Psychiatric Classification Scheme and Diagnostic Criteria (20) were also applied. (2) Interventions: participants in the experimental group received a combination of acupuncture and rTMS. (3) Control Group: control participants were administered either rTMS alone or in combination with conventional Western medicine. (4) Outcome Measures: primary outcome measures included the Hamilton Depression Scale (HAMD) and the Self-Depression Scale (SDS). Secondary outcomes assessed were clinical efficacy, scores on Chinese medicine symptomatology, the Pittsburgh Sleep Quality Inventory (PSQI), the National Institutes of Health Stroke Scale (NIHSS), cognitive function as evaluated by the Mini-Mental State Examination (MMSE) and the Montreal Cognitive Assessment (MoCA), and the Activities of Daily Living Scale (ADL). Inflammatory markers such as interleukin 6 (IL-6), tumor necrosis factor alpha (TNF-α), interleukin 1β (IL-1β), along with neurotransmitters like 5-hydroxytryptamine (5-HT) and brain-derived neurotrophic factor (BDNF), were also measured. (5) Study Type: the study included RCTs, whether blinded or unblinded, published in either Chinese or English.

### 2.3 Literature exclusion criteria

(1) Non-randomized controlled studies, such as literature reviews and case reports; (2) duplicate publications of data from the same trial; (3) studies with incomplete original data; (4) animal studies; and (5) studies lacking primary outcome measures.

### 2.4 Literature screening and data extraction

Literature was screened and verified according to the predefined inclusion and exclusion criteria by two independent researchers. The process included reviewing titles, abstracts, and full texts to identify studies that met the criteria. Discrepancies were resolved through discussion between the two reviewers or, if necessary, consultation with a third party. Data extraction was performed using a standardized Excel template, capturing details such as author names, year of publication, study characteristics, interventions used, and outcome measures.

### 2.5 Quality evaluation

The methodological quality of included studies was evaluated using the Cochrane Collaboration’s Risk of Bias Tool. This tool assesses various factors including random sequence generation, allocation concealment, blinding of participants and personnel, blinding of outcome assessment, completeness of outcome data, selective reporting, and other potential biases. Each factor was classified as high, low, or unclear risk. Two independent researchers conducted these assessments, with any disagreements resolved through discussion or a third-party consultation.

The Modified Jadad Rating Scale (21) was also employed to assess study quality, focusing on random sequence generation, randomization concealment, double-blinding, and the reporting of withdrawals and dropouts. Studies were scored on a scale from 0 to 7, with scores of 3 or lower indicating low quality and scores of 4 or higher indicating high quality.

### 2.6 Evidence quality evaluation

The GRADE profiler 3.6 tool was utilized to evaluate the quality of evidence, which is categorized into four levels: high (A), medium (B), low (C), and extremely low (D). During the evaluation process, the decision to downgrade the evidence was based on five key factors: research limitations, inconsistency, indirectness, imprecision, and publication bias.

### 2.7 Statistical methods

Meta-analyses were conducted using Review Manager (RevMan) 5.4. Outcome measures were presented as odds ratios (OR) or risk ratios (RR) for dichotomous data, and mean differences (MD) or standardized mean differences (SMD) for continuous data. Confidence intervals (CIs) were calculated at the 95% level. Heterogeneity was assessed using the *I*^2^ statistic; a fixed-effects model was applied when *p* > 0.10 and *I*^2^ ≤ 50%, while a random-effects model was used for *p* ≤ 0.10 and *I*^2^ > 50%. Subgroup or sensitivity analyses were performed as needed. Publication bias was evaluated using funnel plots when the analysis included more than ten outcomes.

## 3 Results

### 3.1 Literature screening

**Figure 1:**
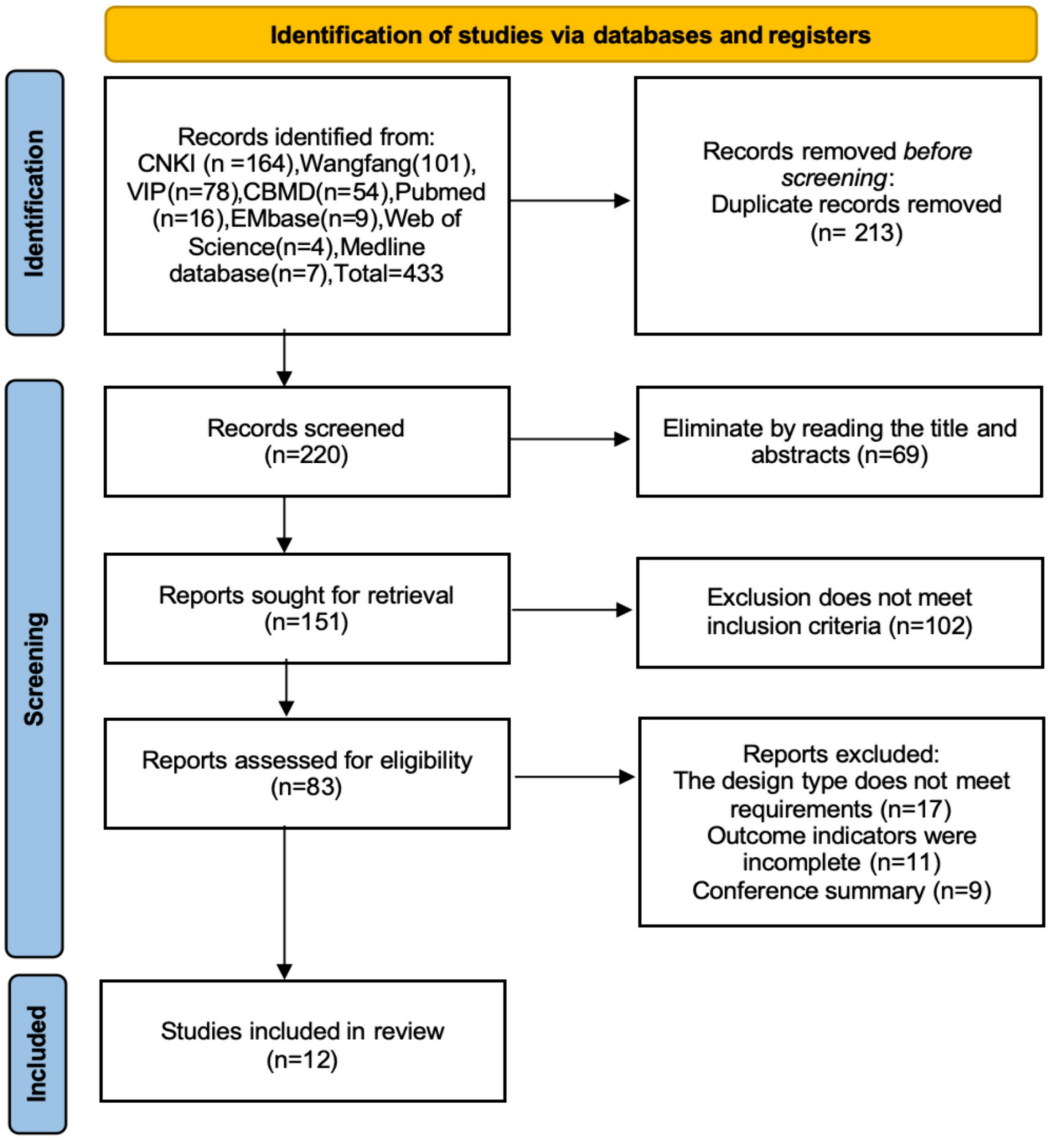
Literature screening process for acupuncture combined with rTMS in the treatment of PSD.

In total, 433 literatures were retrieved. After removing duplicates using Endnote software, 220 literatures remained. Following the application of inclusion and exclusion criteria, 12 literatures were ultimately selected for inclusion (22–33). The literature screening process is illustrated in Figure 1.

### 3.2 Basic information on included studies

Twelve RCTs involving 800 patients were included, comprising 400 patients in the treatment group and 400 in the control group. The baseline characteristics of the studies were comparable, detailed in Table 1.

**Table 1:** Basic characteristics of the included literature.

Study	Case		Mean age (year)		Intervention		Disease course (mouth)		Time (week)	Outcomes
	T(F/M)	C(F/M)	T	C	T	C	T	C		
Hu (22)	30 (12/18)	30 (16/14)	50 ± 9	53 ± 10	Acupuncture + rTMS + CWM	Acupuncture + CWM	5.15 ± 3.17	5.78 ± 3.04	4	②⑥
Liu (23)	30 (19/11)	30 (18/12)	66.63 ± 11.39	65.95 ± 10.73	Acupuncture + rTMS + CWM	Acupuncture + CWM	2.02 ± 1.2	2.11 ± 1.08	8	①②④⑤⑥
Meng (24)	39 (27/12)	39 (25/14)	59.18 ± 6.2	60.37 ± 6.12	Acupuncture + rTMS + CWM	rTMS + CWM	3.59 ± 0.37	3.62 ± 0.41	4	①②④⑨
Cui (25)	32 (19/13)	32 (20/12)	65.25 ± 5.06	65.98 ± 5.21	Acupuncture + rTMS + CWM	rTMS + CWM	2.24 ± 1.04	2.67 ± 1.53	4	①②⑨⑩
Qin (26)	40 (22/18)	40 (25/15)	61.24 ± 2.21	62.03 ± 1.41	Acupuncture + rTMS + CWM	rTMS + CWM	3.97 ± 0.21	3.93 ± 0.26	4	①③⑤⑦⑨
Zhang (27)	30 (17/13)	30 (18/12)	58.2 ± 10.32	59.3 ± 10.83	Acupuncture + rTMS + CWM	rTMS + CWM	3.5 ± 0.56	3.73 ± 0.45	12	①②⑤⑥⑨
Yin (28)	30 (17/13)	30 (20/10)	57.61 ± 7.81	56.13 ± 6.92	Acupuncture + rTMS + CWM	rTMS + CWM	4.15 ± 0.94	3.94 ± 1.12	4	②④⑥⑨
Zhang (29)	48 (27/21)	48 (21/27)	61.35 ± 7.41	62.2 ± 6.79	Acupuncture + rTMS + CWM	rTMS + CWM	2.50 ± 2.90	2.43 ± 2.83	4	①②⑦⑧⑨⑩
Niu (30)	30 (18/12)	30 (10/20)	53.72 ± 7.41	52.1 ± 6.79	Acupuncture + rTMS + CWM	rTMS + CWM	1.01 ± 2.28	1.96 ± 2.89	4	①②⑥⑧
Niu (31)	40 (28/12)	40 (13/27)	64.01 ± 6.73	63.79 ± 6.79	Acupuncture + rTMS + CWM	rTMS + CWM	–	–	6	①③⑧⑩
Chen (32)	25 (14/11)	25 (10/15)	67.45 ± 7.38	66.19 ± 6.97	Acupuncture + rTMS + CWM	rTMS + CWM	3.89 ± 2.17	4.11 ± 1.98	12	①③⑥⑦⑨
Tan (33)	26 (13/13)	26 (16/10)	61.37 ± 3.20	62.54 ± 2.97	Acupuncture + rTMS + CWM	rTMS + CWM	–	–	12	①②④⑤

T, Experimental group; C, Control group; −, Not mentioned; CWM, Conventional western medicine; ①. Clinical effective rate; ②, HAMD score; ③, SDS score; ④, Cognitive function (MMSE, MoCA); ⑤, ADL score; ⑥, PSQI score; ⑦, TCM syndrome score; ⑧, NIHSS; ⑨, Neurotransmitters (5-HT, BDNF); ⑩, Inflammatory cytokines (TNF-α,1 L-1β, IL-6).

### 3.3 Quality assessment of included studies

**Figure 2:**
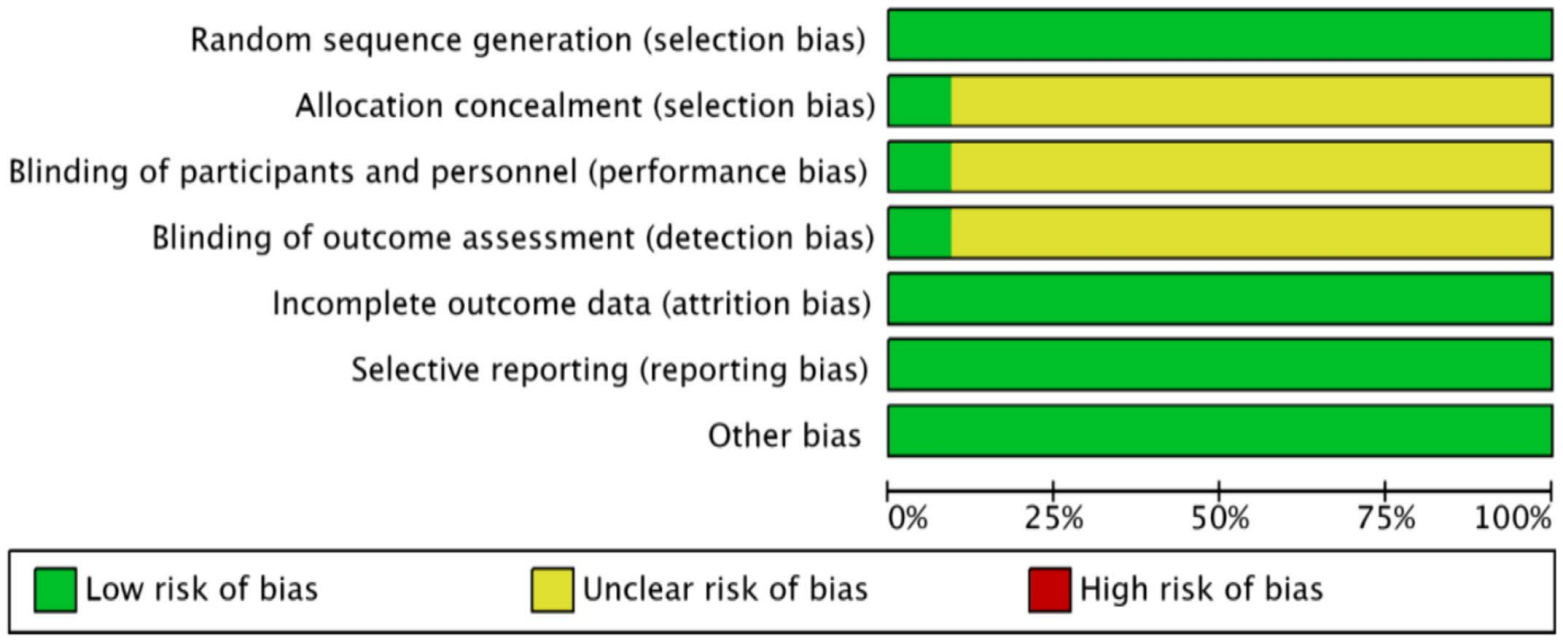
Acupuncture combined with rTMS for the treatment of depressive states after stroke proportion of risk of bias for inclusion in the literature.

**Figure 3:**
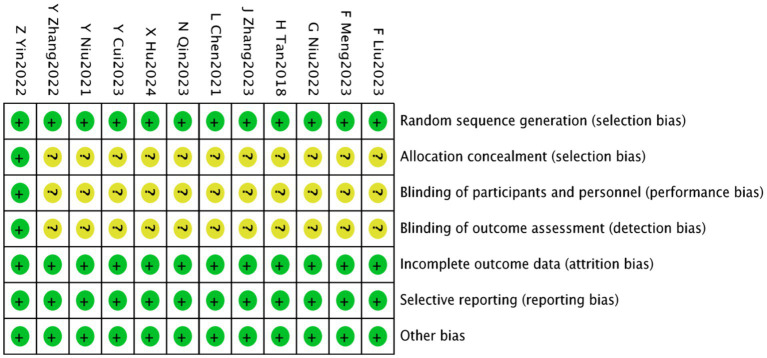
Acupuncture combined with rTMS for the treatment of depressive states after stroke a methodological quality assessment of the included literature.

Eleven studies (22–33) employed the random number table method for randomisation. Only one study (28) reported concealed allocation and employed a double-blind methodology, thus it was assessed as low risk. The remaining ten studies did not specify these details and were therefore categorized as having unclear risk. All included studies provided complete outcome data with no evidence of selective reporting or other biases, and were thus assessed as low risk. These assessments are depicted in Figures 2, 3. Details of the Jadad scores for the included studies are provided in Supplementary Table S2.

### 3.4 Meta-analysis results

#### 3.4.1 Clinical effectiveness rate

**Figure 4:**
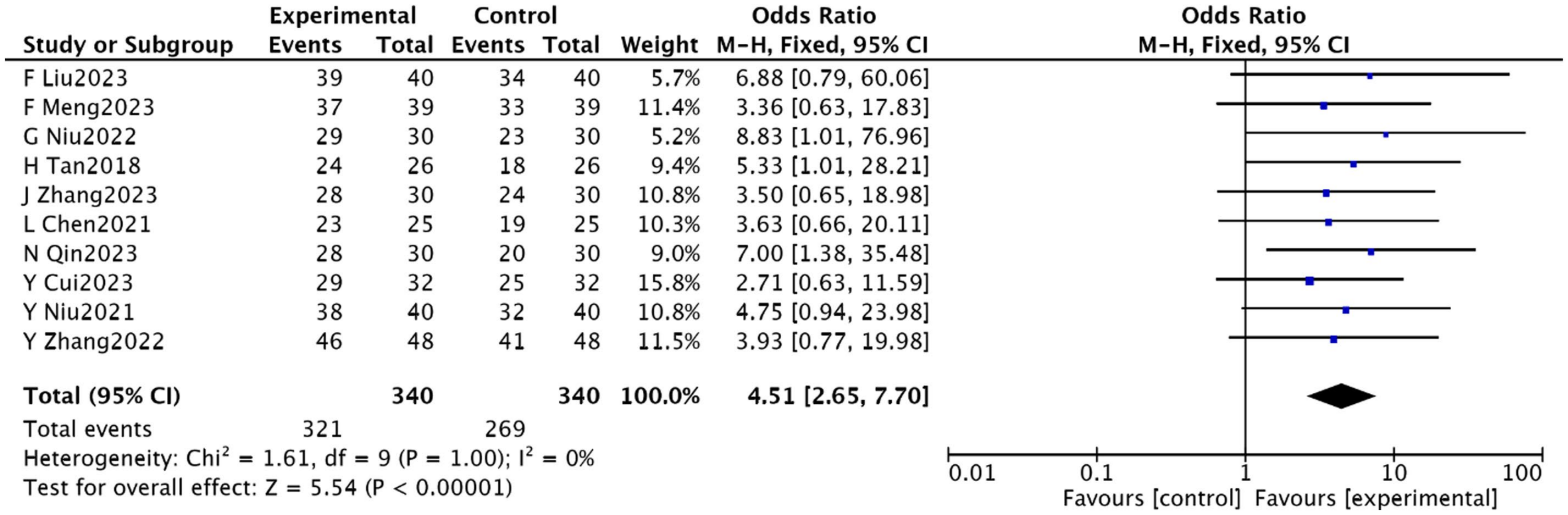
Meta-analysis of the effectiveness of acupuncture combined with rTMS in the treatment of PSD.

Ten studies (23–27, 29–33), involving 680 patients, reported on clinical efficacy. These studies showed homogeneity (*p* = 0.90, *I*^2^ = 0), permitting a fixed-effect meta-analysis. The results indicated a significantly higher clinical effectiveness rate in the experimental group compared to the control group [RR = 1.19, 95% CI (1.12, 1.27), *p* < 0.00001], as illustrated in Figure 4.

#### 3.4.2 HAMD scores

**Figure 5:**
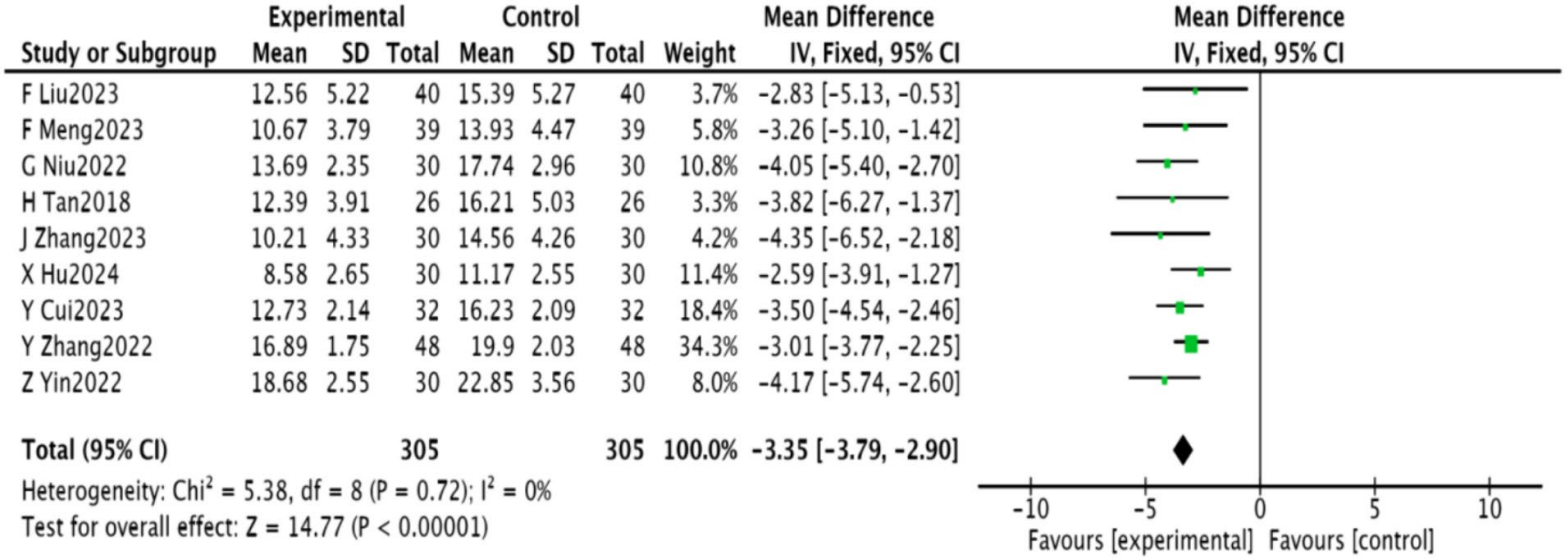
Meta-analysis of HAMD scores in acupuncture combined with rTMS for PSD.

Nine studies (22–25, 27, 29, 30, 32) involving 710 patients reported on HAMD scores. Homogeneity was observed (*p* = 0.72, *I*^2^ = 0), enabling a fixed-effect meta-analysis. There was a significant reduction in HAMD scores in the experimental group [MD = −3.35, 95% CI (−3.79, −2.90), *p* < 0.00001], shown in Figure 5.

#### 3.4.3 SDS scores

**Figure 6:**
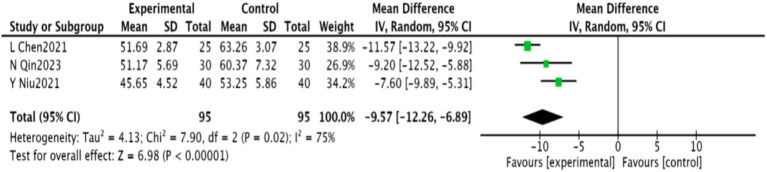
Meta-analysis of SDS scores in acupuncture combined with rTMS for PSD.

Three studies (25, 30, 31), including 190 patients, reported SDS scores. These studies showed significant heterogeneity (*p* = 0.02, *I*^2^ = 75%), necessitating a random-effects meta-analysis. A significant reduction in SDS scores was found in the experimental group [MD = −9.57, 95% CI (−12.26, −6.89), *p* < 0.00001], as shown in Figure 6.

#### 3.4.4 Chinese medicine symptom score

**Figure 7:**
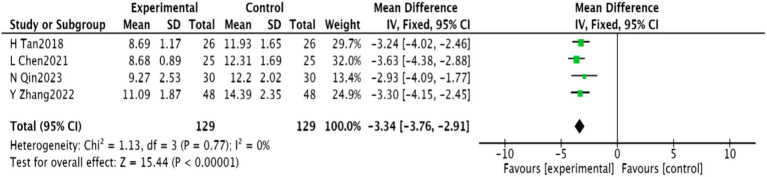
Meta-analysis of Chinese medicine symptom scores in acupuncture combined with rTMS for PSD.

Four studies (25, 27, 28, 31) involving 258 patients reported on Traditional Chinese Medicine (TCM) symptom scores. These studies demonstrated homogeneity (*p* = 0.77, *I*^2^ = 0), and a fixed-effect meta-analysis showed a significant decrease in TCM symptom scores [MD = −3.34, 95% CI (−3.76, −2.91), *p* < 0.00001], illustrated in Figure 7.

#### 3.4.5 PSQI score

**Figure 8:**
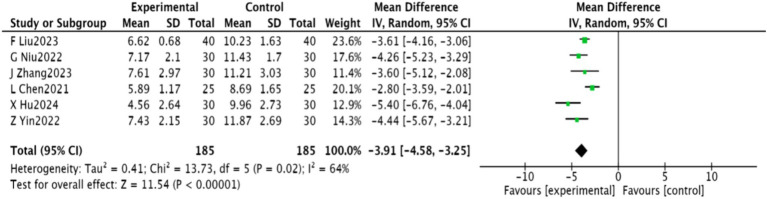
Meta-analysis of PSQI scores in acupuncture combined with rTMS for PSD.

Six studies (22, 27, 28, 30, 32, 33) involving 370 patients reported PSQI scores. These studies were heterogeneous (*p* = 0.02, *I*^2^ = 64%) and a random-effects meta-analysis indicated a significant improvement in PSQI scores [MD = −3.91, 95% CI (−4.58, −3.25), *p* < 0.00001]. The results are depicted in Figure 8.

#### 3.4.6 NIHSS score

**Figure 9:**
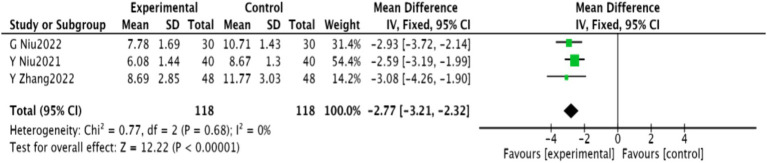
Meta-analysis of NIHSS scores in acupuncture combined with rTMS for PSD.

Three studies (28–30), comprising 236 patients, reported NIHSS scores. Homogeneity was noted (*p* = 0.68, *I*^2^ = 0), and a fixed-effect meta-analysis revealed a significant reduction in NIHSS scores [MD = −2.77, 95% CI (−3.21, −2.32), *p* < 0.00001], shown in Figure 9.

#### 3.4.7 Cognitive functions

**Figure 10:**
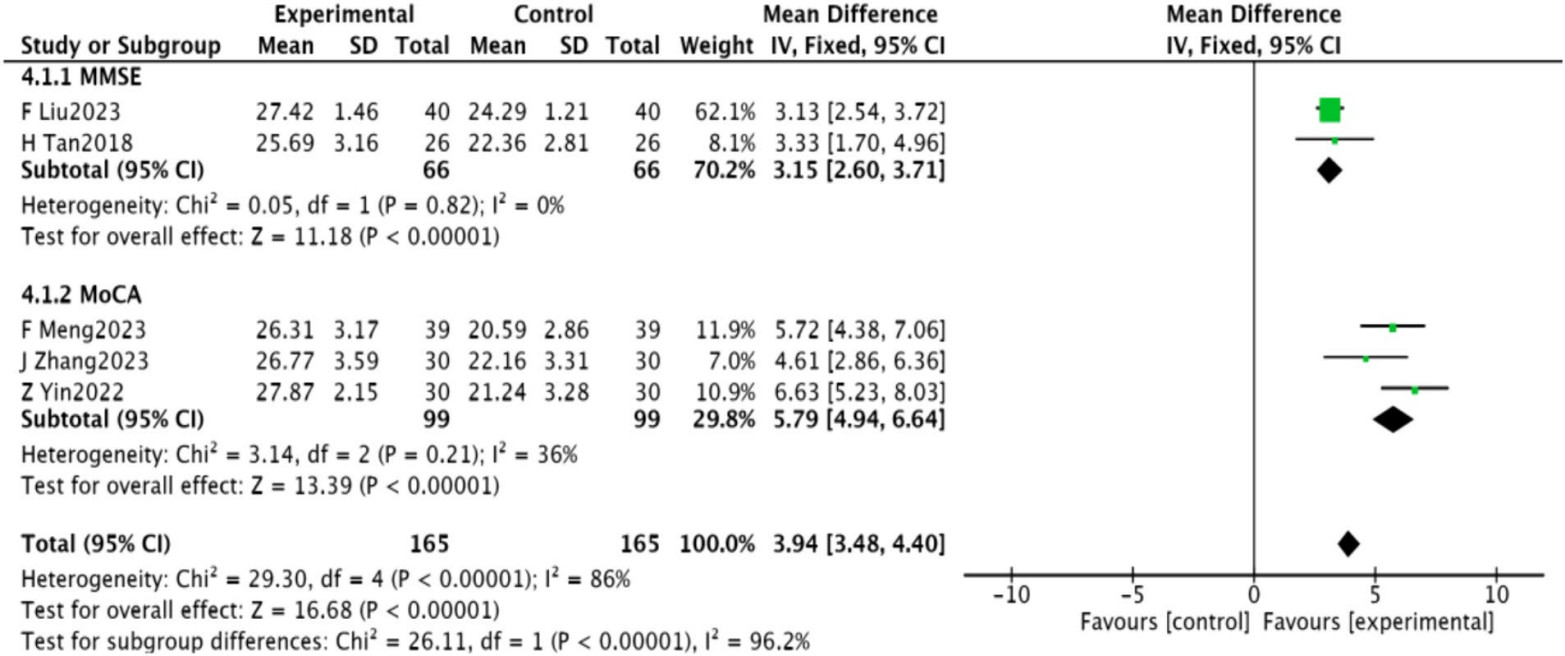
Meta-analysis of cognitive function in acupuncture combined with repetitive transcranial magnetic stimulation for PSD.

MMSE scores were reported in two studies (22, 32) involving 132 patients, demonstrating homogeneity (*p* = 0.82, *I*^2^ = 0) and enabling a fixed-effect meta-analysis. A significant improvement in MMSE scores was observed [MD = 3.15, 95% CI (2.60, 3.71), *p* < 0.00001]. Additionally, MoCA scores were reported in two studies (23, 26) involving 198 patients, with similar homogeneity (*p* = 0.21, *I*^2^ = 36%). A significant increase in MoCA scores was noted [MD = 5.79, 95% CI (4.94, 6.64), *p* < 0.00001], as detailed in Figure 10.

#### 3.4.8 Inflammatory factors

**Figure 11:**
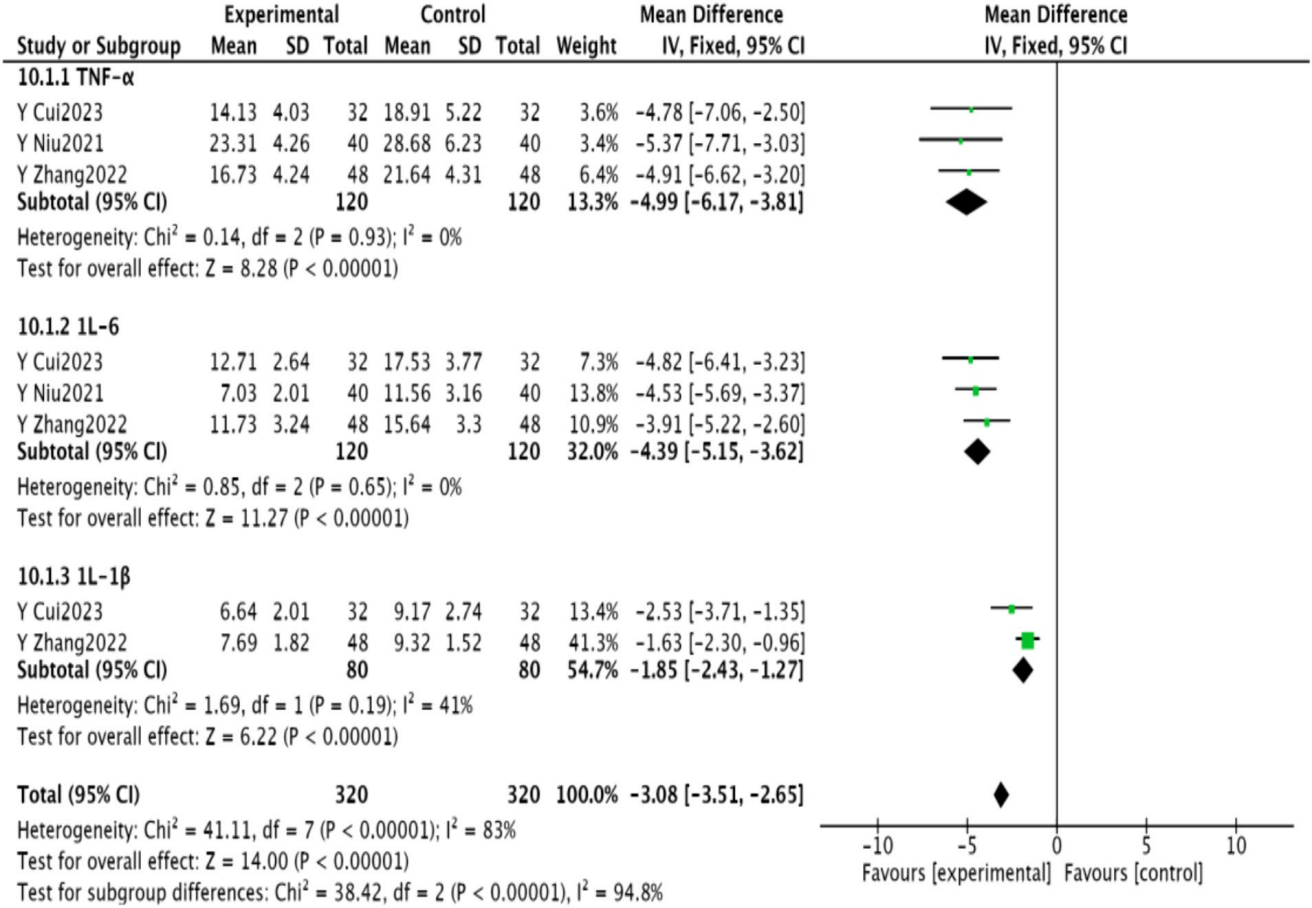
Meta-analysis of inflammatory factors in PSD after acupuncture combined with repetitive transcranial magnetic stimulation in WisdomLink stroke.

Three studies (24, 27, 30) reported on IL-6 and TNF-α levels, while two studies (24, 30) reported on IL-1β. A meta-analysis revealed significantly lower levels of these markers in the experimental group (*p* < 0.00001). These findings are presented in Figure 11.

#### 3.4.9 Neurotransmitters

**Figure 12:**
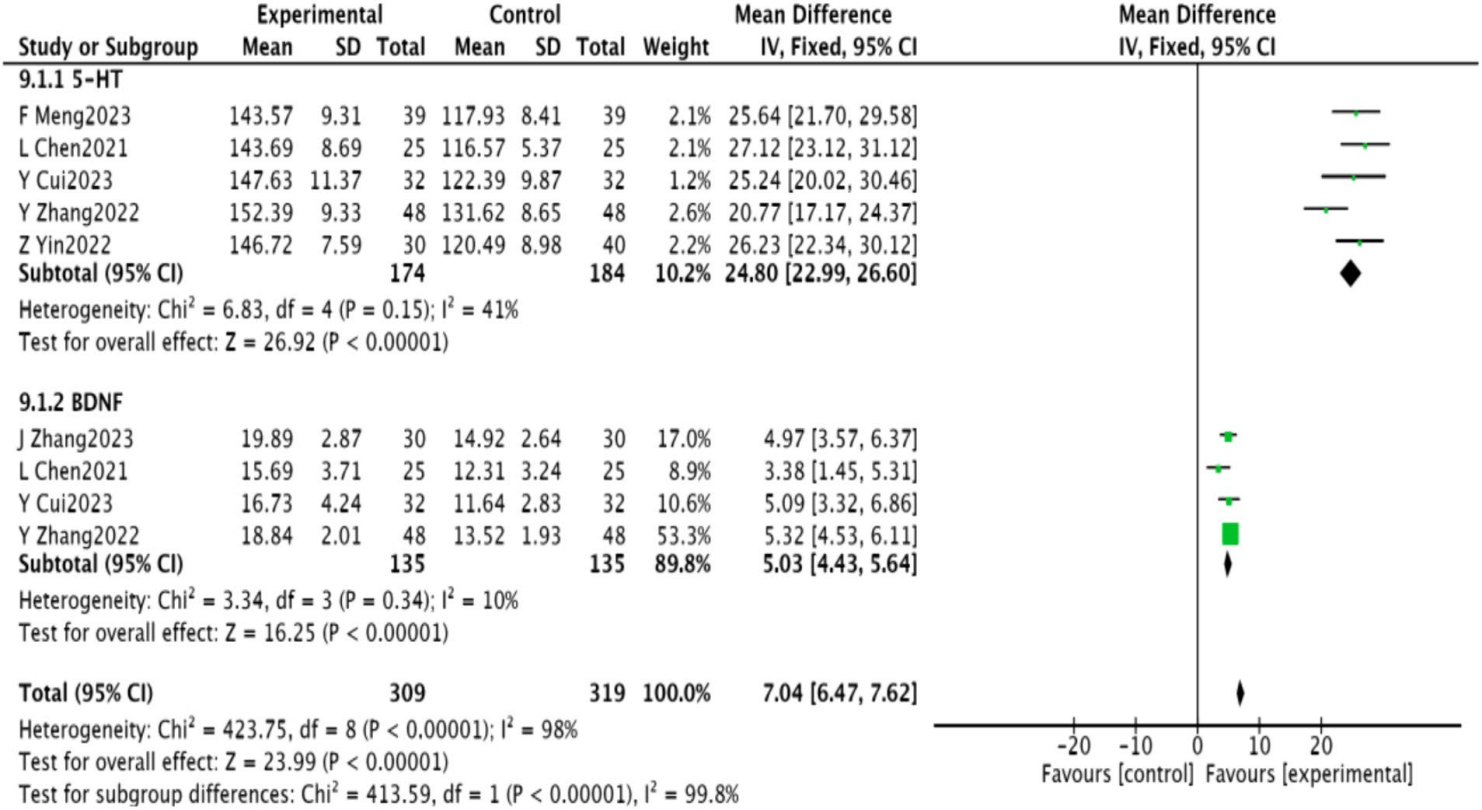
Neurotransmitter meta-analysis of depression after acupuncture combined with repetitive transcranial magnetic stimulation in wise link stroke.

Five studies (24–27, 30) reported on 5-HT levels, while four studies (24, 27, 29, 31) reported on BDNF. Meta-analysis indicated significantly higher levels of 5-HT and BNF in the experimental group (*p* < 0.00001). The results are detailed in Figure 12.

#### 3.4.10 Risk of publication bias assessment

**Figure 13:**
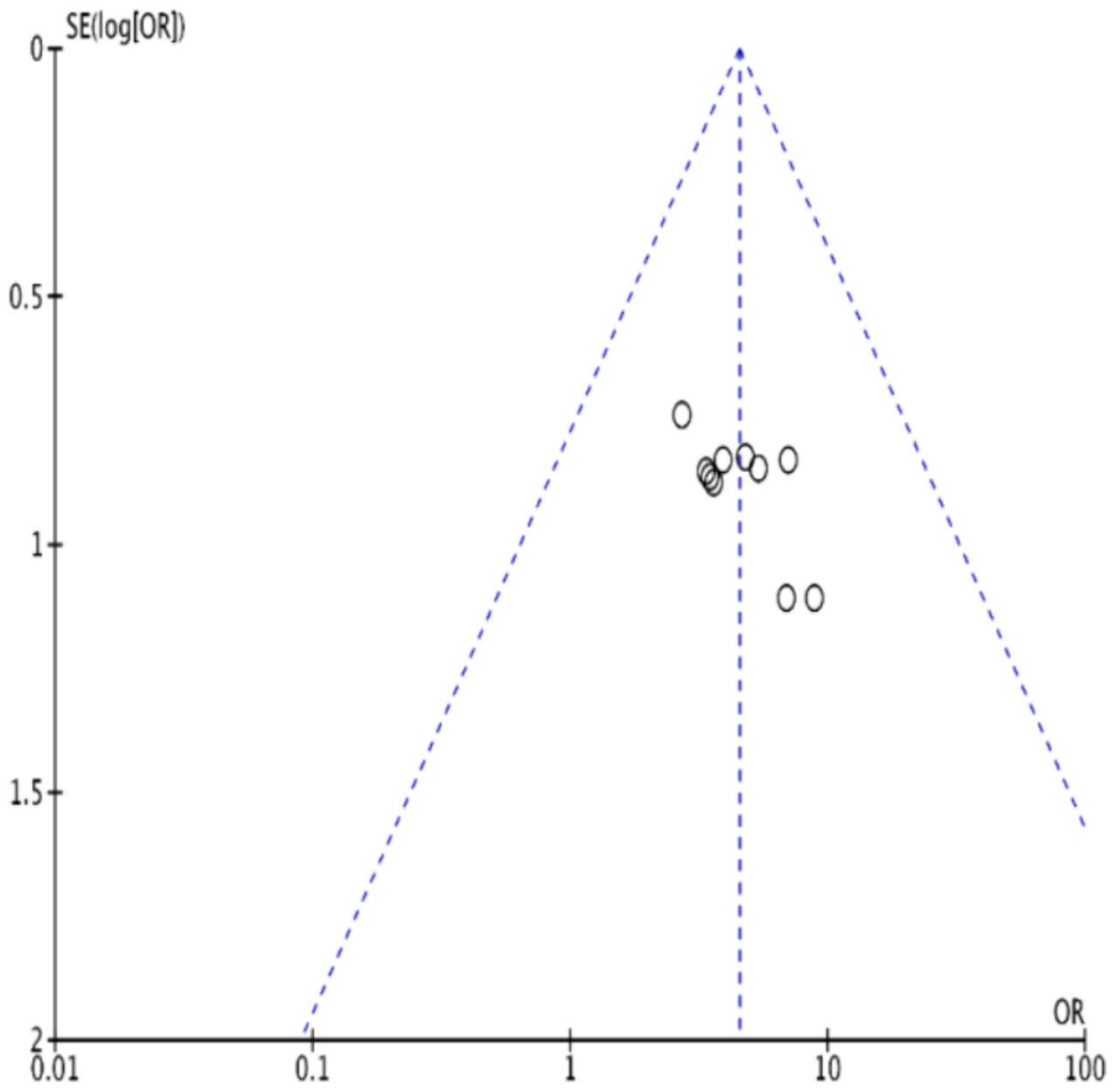
Funnel plot of clinical effectiveness of acupuncture combined with repetitive transcranial magnetic stimulation in the treatment of PSD.

**Figure 14:**
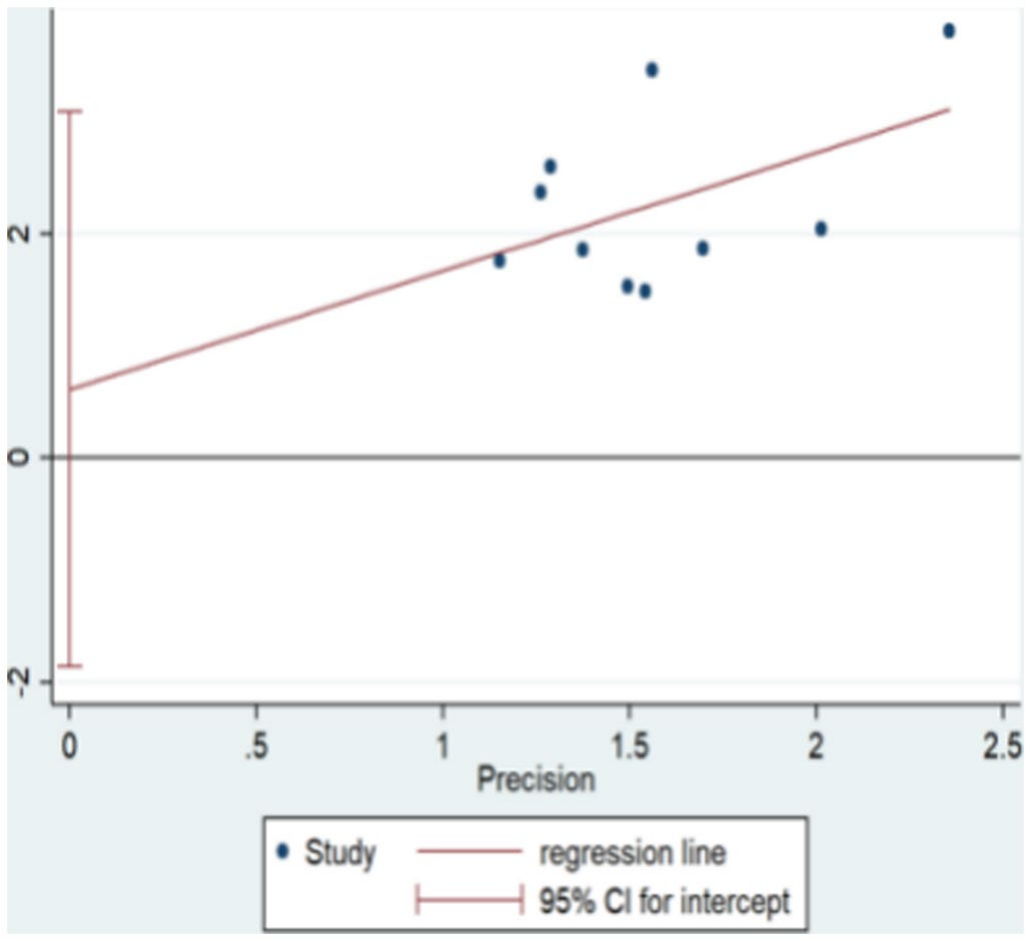
The efficient Eggers test.

The funnel plot for clinical validity suggests approximate symmetry, indicating minimal publication bias. This is visually represented in Figure 13. Further analysis using Egger’s test shows an even distribution of study points on both sides of the line, yielding a *p*-value of 0.583, which suggests no significant publication bias. This is visually represented in Figure 14.

### 3.5 Evidence quality evaluation

The results of the GRADE assessment, which evaluates the evidence for the effectiveness of combining acupuncture with rTMS in treating PSD, can be found in Supplementary Table S3. The primary reasons for downgrading were inadequate blinding and insufficient allocation concealment. Significant heterogeneity was observed in some studies without reasonable explanation, impacting the methodological rigour and reliability of the results. Additionally, imprecision required downgrading due to broad confidence intervals, affecting the precision of the evidence.

## 4 Discussion

Post-stroke residual trunk dysfunction and decreased language function are commonly addressed in clinical treatment; however, psycho-emotional changes such as depression are frequently overlooked. While the precise causes of PSD are not fully understood, existing research suggests that a combination of neurobiological factors and psychosocial aspects of stroke contribute to its development (34–37). PSD not only disrupts normal neurological functions but also prolongs recovery, reduces self-care capabilities, and can lead to extreme behaviours such as suicide and self-harm (38).

In Chinese medicine, PSD is categorized as an “affective disease” and falls under the domain of “depression evidence.” It is thought to arise from deficiencies in liver and kidney function, insufficient qi and blood, dietary imbalances, as well as emotional and psychological trauma. These deficiencies disrupt the flow of qi and blood, exacerbating post-stroke conditions and impairing the functionality of internal organs. The liver’s compromised ability to detoxify and regulate qi often manifests as depressive syndromes (39). Typically, acupuncture treatments for PSD focus on enhancing mental clarity, opening orifices, regulating liver function, and promoting the smooth flow of qi. Additional acupoints that strengthen the liver and kidney, activate blood circulation, and resolve blood stasis are also frequently employed (40). Experimental research has demonstrated that acupuncture and moxibustion can regulate the 5-HT system and the functionality of 5-HT1A receptors in depression model rats (41). Acupuncture has been shown to elevate levels of tryptophan and 5-HT in peripheral serum, enhance tryptophan in the blood and cerebrospinal fluid, and promote 5-HT synthesis in the brain (42). Additionally, it can increase peripheral serum BDNF levels and mitigate neuropathic diseases by modulating the BDNF signalling pathway, thus enhancing 5-HT synthesis in the brain (43).

The prevailing Western medical approach to PSD primarily employs pharmacological interventions, supplemented by non-pharmacological treatments. Common medications include benzodiazepines, tricyclic antidepressants, SSRIs, and SNRIs (3). However, the long-term use of antidepressants, potential for dependency, and various side effects have led to an increased focus on non-pharmacological options (44). These include psychotherapy, cognitive therapy, rTMS, tDCS, electroencephalographic biofeedback therapy, and TCM rehabilitation. rTMS, as a novel technology in targeted post-stroke rehabilitation, aims to stimulate or inhibit specific brain areas by adjusting stimulation parameters, thereby modulating cortical activity and facilitating functional mapping of corresponding cortical regions (45). The therapeutic potential of rTMS may be related to increased BDNF concentration, enhanced glucose metabolism, neuroplasticity, and modulation of neural biochemical effects within the cerebral cortex and specific neural networks (46).

In clinical practice, the emphasis is increasingly on the implementation of integrative treatment modalities for managing PSD. This approach seeks to discover more effective and safer therapeutic strategies (47, 48). In recent years, integrative medicine—an emergent medical system—has gained recognition and support within the community (49). By synergistically merging advanced theoretical knowledge with clinical practice and adapting it to consider social, psychological, and environmental factors, integrative medicine is positioned as the inevitable evolution of future medical practice, particularly from a holistic human health perspective.

Combining acupuncture with rTMS offers a reliable, safe, and well-tolerated physiotherapeutic approach for treating patients with PSD. Currently, studies and systematic evaluations examining the use of acupuncture and rTMS for PSD are limited. rTMS application in China is primarily conducted in major tertiary hospitals, with few institutions employing both acupuncture and rTMS for treating PSD. This study conducted a meta-analysis and systematic review to assess the clinical efficacy and safety of combining acupuncture with rTMS for treating PSD, drawing on RCTs conducted both nationally and internationally. The aim is to offer clinicians a safe and effective alternative treatment.

This study analysed 12 papers involving 800 patients. The meta-analysis indicated that integrating acupuncture with rTMS therapy could enhance clinical efficacy in patients with PSD. It exhibited superior effects on TCM symptom scores, HAMD scores, SDS scores, NIHSS scores, ADL scores, PSQI scores, cognitive functions (MMSE and MoCA scores), inflammatory factors (IL-6, TNF-α, IL-1β), and neurotransmitter levels (5-HT, BDNF) compared to the control group, thereby confirming its efficacy. Regarding safety, the study demonstrated that the combined use of acupuncture and rTMS is effective in treating PSD and merits further promotion.

However, this study has several limitations. Among the 12 included studies, only one reported allocation concealment and blinding, while the remaining 11 did not, leading to generally low-quality articles. Additionally, the study protocols and sample size estimations were not reported, suggesting a lack of rigour in clinical trial design and consequently reducing the reliability of the study results. Moreover, all 12 studies were conducted in China, introducing potential geographical bias. Therefore, the conclusions of this study require validation through additional high-quality research.

## Data availability statement

The raw data supporting the conclusions of this article will be made available by the authors, without undue reservation.

## Author contributions

KX: Writing – original draft, Writing – review & editing. XL: Data curation, Writing – review & editing, Software, Writing – original draft. WH: Data curation, Formal analysis, Methodology, Writing – review & editing, Writing – original draft. XhL: Funding acquisition, Supervision, Writing – review & editing, Writing – original draft.
